# Accumulation of Radioactive Cesium Released from Fukushima Daiichi Nuclear Power Plant in Terrestrial Cyanobacteria *Nostoc commune*

**DOI:** 10.1264/jsme2.ME13035

**Published:** 2013-11-19

**Authors:** Hideaki Sasaki, Susumu Shirato, Tomoya Tahara, Kenji Sato, Hiroyuki Takenaka

**Affiliations:** 1 College of Science and Engineering, Iwaki Meisei University, 5–5–1 Chuodai Iino, Iwaki, Fukushima 970–8551, Japan; 2 Iwaki Community Reconstruction Center, Iwaki Meisei University, 5–5–1 Chuodai Iino, Iwaki, Fukushima 970–8551, Japan; 3 MAC Gifu Institute, Microalgae Corporation, 4–15 Akebono-cho, Gifu 500–8148, Japan

**Keywords:** *Nostoc commune*, terrestrial cyanobacteria, radioactive cesium, Fukushima Daiichi Nuclear Power Plant

## Abstract

The Fukushima Daiichi Nuclear Power Plant accident released large amounts of radioactive substances into the environment and contaminated the soil of Tohoku and Kanto districts in Japan. Removal of radioactive material from the environment is an urgent problem, and soil purification using plants is being considered. In this study, we investigated the ability of 12 seed plant species and a cyanobacterium to accumulate radioactive material. The plants did not accumulate radioactive material at high levels, but high accumulation was observed in the terrestrial cyanobacterium *Nostoc commune*. In Nihonmatsu City, Fukushima Prefecture, *N. commune* accumulated 415,000 Bq/kg dry weight ^134^Cs and 607,000 Bq kg^−1^ dry weight ^137^Cs. The concentration of cesium in *N. commune* tended to be high in areas where soil radioactivity was high. A cultivation experiment confirmed that *N. commune* absorbed radioactive cesium from polluted soil. These data demonstrated that radiological absorption using *N. commune* might be suitable for decontaminating polluted soil.

Radioactive material was released into the environment in large quantities after the accident at the Fukushima Daiichi Nuclear Power Plant in March 2011, contaminating the soils of Tohoku and Kanto districts in Japan. According to the calculations of the Tokyo Electric Power Company, approximately 9.0×10^17^ Bq of radioactive material was released by the accident (14). The level of this radioactive material is being monitored (1, 12), and its accumulation by and effects on organisms have been reported (3, 4).

Plants are primary producers and can accumulate radioactive material. Contamination of crops and wild plants is thus a major concern (7, 9, 13), but the details are poorly understood. For example, a sunflower that accumulated high levels of radioactive material was used to purify soil polluted after the Chernobyl Nuclear Power Plant accident that occurred in 1986 in Russia (2, 5, 10). However, a decontamination experiment using a sunflower performed by the Ministry of Agriculture, Forestry and Fisheries of Japan in 2011 was not successful (6).

While investigating the accumulation of radioactive material in wild plants and cyanobacterium in Iwaki City, Fukushima Prefecture, Japan, high accumulation was observed in the terrestrial cyanobacterium *Nostoc commune*. This species is a heterocystous blue-green algae that forms jelly-like clumps of polysaccharides. Radioactive material is expected to be deposited in these polysaccharides, simplifying its removal from the environment (11). Furthermore, it is reported that this species grows under high radiation exposure (8). In this study, we monitored the accumulation of radioactive material in *N. commune*.

## Materials and Methods

### Collection of seed plants and cyanobacterium *N. commune*

**Fig. 1:**
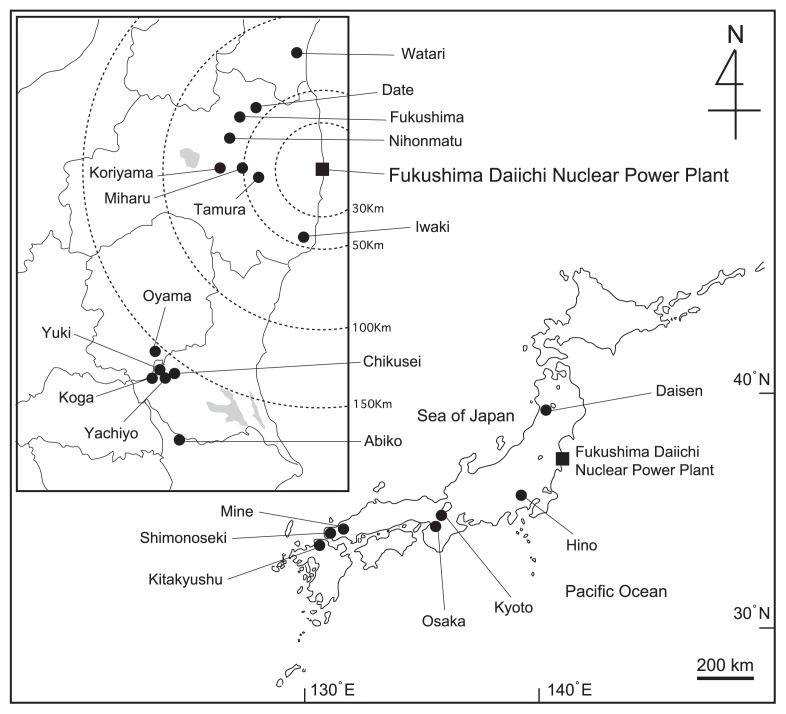
Locations of the collection sites of the *Nostoc commune* investigated in the present study.

Twelve species of seed plants and cyanobacterium *N. commune* were collected in 2012 from a site belonging to Iwaki Meisei University in Fukushima Prefecture, Japan. The site is approximately 45 km from the Fukushima Daiichi Nuclear Power Plant. *N. commune* was also collected from 34 habitats in Akita, Miyagi, Fukushima, Ibaraki, Tochigi, Chiba, Tokyo, Kyoto, Osaka, Yamaguchi, and Fukuoka prefectures, Japan, in 2011 and 2012 (Fig. 1).

### Detection of radioactive cesium and iodine

Field materials collected were transported to the laboratory in a polyethylene bag at 15–20°C to avoid damage during transportation. Plant and cyanobacterial samples were washed with water and dried at room temperature. Dried samples were desiccated at 60°C for 48 h, pulverized using a mill, and placed in U-8 plastic vials (Φ56 mm×68 mm high). Vials were stored in a desiccator until measurement. Radioactive cesium (^134^Cs, ^137^Cs) and iodine (^131^I) were measured using a GEM40P4-76 Ge semiconductor detector (Seiko EG & G, Tokyo, Japan). Soil samples were dried at 60°C for 48 h, sifted through a Φ2.8 mm mesh, and placed in V-11 plastic vials (Φ128 mm×76 mm high). Levels of ^134^Cs, ^137^Cs, and ^131^I were measured using a CAN-OSP-NAI NaI scintillation counter (Hitachi-Aloka Medical, Tokyo, Japan) or a Ge semiconductor detector.

### Cultivation experiments

We used *N. commune* cultivated on Miyakojima Island, Okinawa, Japan, for this experiment. Dried *N. commune* (30 g) was soaked in distilled water for 1 h and then put on the surface of polluted sand or loam soil (1 kg) in a plastic box (40 cm length×32 cm width×7 cm height). The samples were cultivated outdoors for 30 days in November 2011. The concentration factor was calculated using the formula (radioactive cesium concentration in *N. commune*)/(radioactive cesium concentration in soil), where the cesium concentration was measured in Bq kg^−1^ dry weight (DW).

## Results and Discussion

We investigated soil contamination by radioactive material at a site of Iwaki Meisei University. In April 2012, the radioactivity was distributed within the top 10 cm of topsoil (Table 1). The concentration of ^137^Cs was 2,530 Bq kg^−1^ DW at depths of 0–5 cm, 424 Bq kg^−1^ DW at 5–10 cm, 31 Bq kg^−1^ DW at 10–15 cm, and 5 Bq kg^−1^ DW at 15–20 cm. ^131^I was not detected, perhaps because of its short half-life (8.02 days).

In all the 12 seed plant species studied, we detected relatively low concentrations of radioactive cesium (Table 2). Accumulation levels in larger plants such as *Solidago canadensis* var. *scabra* tended to be lower than in smaller plants. In *S. canadensis* var. *scabra*, the concentrations of ^137^Cs were 78 Bq kg^−1^ DW in shoots and 100 Bq kg^−1^ DW in roots. In contrast, the small plant *Vicia sativa* ssp. *nigra* accumulated 557 Bq kg^−1^ DW ^137^Cs in shoots and 1,520 Bq kg^−1^ DW in roots. We believe that *V. sativa* ssp. *nigra* absorbed more radioactive cesium because of its small root system. Overall, more radioactive material accumulation was concentrated in the roots than in the shoots. In the shoots, ^134^Cs levels were 29–410 Bq kg^−1^ DW and ^137^Cs levels were 43–557 Bq kg^−1^ DW. In the roots, ^134^Cs levels were not detected (ND) to 1,120 Bq kg^−1^ DW and ^137^Cs levels were ND–1,520 Bq kg^−1^ DW. These data suggested that seed plants generally do not easily transport radioactive cesium into shoots. In contrast, high concentrations of ^134^Cs (32,300 Bq kg^−1^ DW) and ^137^Cs (46,200 Bq kg^−1^ DW) were observed in terrestrial cyanobacterium *N. commune*.

*N. commune* was collected from 34 habitats between August 2011 and October 2012. The radioactivity concentrations of ^134^Cs and ^137^Cs are summarized in supplementary Table 1. The ^134^Cs concentrations ranged from not detectable (ND) to 415,000 Bq kg^−1^ DW, while the ^137^Cs concentrations ranged from ND to 607,000 Bq kg^−1^ DW. Radioactive iodine ^131^I was not detected in any samples. High radioactive cesium levels were observed in the samples from Fukushima Prefecture, where the Fukushima Daiichi Nuclear Power Plant is located. In particular, 415,000 Bq kg^−1^ DW ^134^Cs and 607,000 Bq kg^−1^ DW ^137^Cs were detected in *N. commune* from Nihonmatsu, where soil contamination was high (5,460 Bq kg^−1^ DW ^134^Cs, 6,330 Bq kg^−1^ DW ^137^Cs). However, much lower radioactivity levels (28,300 Bq kg^−1^ DW ^134^Cs, 39,800 Bq kg^−1^ DW ^137^Cs) were detected in the cyanobacterial sample from Koriyama, although soil radioactivity was very high there (26,500 Bq kg^−1^ DW ^134^Cs, 33,200 Bq kg^−1^ DW ^137^Cs). *N. commune* grows throughout the year. We believe that the cyanobacteria sampled from Nihonmatsu existed at the time of the nuclear accident.

Relatively high concentrations of cesium were also detected in the samples from Miyagi, Ibaraki, and Chiba prefectures surrounding Fukushima. In Abiko City (Chiba Prefecture), approximately 200 km from the power plant, 5,380 Bq kg^−1^ DW ^134^Cs and 7,590 Bq kg^−1^ DW ^137^Cs were detected in *N. commune*. Abiko City was reported to have high levels of radioactivity in rain after the accident. In contrast, radioactive cesium was not detected in samples from Osaka, Yamaguchi, and Fukuoka prefectures; cesium released after the accident probably did not reach these areas.

The concentration of radioactive cesium in *N. commune* tended to be high where soil contamination was high. Growing on the soil surface, *N. commune* appeared to have been strongly exposed to radioactivity. As shown in supplementary Fig. 1, the radioactive cesium concentrations in the soil and in *N. commune* were directly proportional and relatively well correlated.

*N. commune* might absorb radioactive cesium from polluted soil or contaminated rainwater. To clarify how radioactive cesium was absorbed by *N. commune*, we cultivated the cyanobacterium in polluted sand and loam soils. Radioactive cesium was not detected from *N. commune* cultivated on Miyakojima Island, which was used for the experiment. During the experimental period, there were three rain showers, but radioactive cesium was not detected in rainwater. The temperature ranged 6–25°C. After 30 days, growth of *N. commune* was not observed, but it had absorbed radioactive cesium (Table 3). In polluted sand (4,980 Bq kg^−1^ DW ^134^Cs, 6,070 Bq kg^−1^ DW ^137^Cs), *N. commune* accumulated 3,100 Bq kg^−1^ DW ^134^Cs and 4,420 Bq kg^−1^ DW ^137^Cs. The concentration factors were 0.62 for ^134^Cs and 0.73 for ^137^Cs. Similarly, in polluted loam soil (785 Bq kg^−1^ DW ^134^Cs, 962 Bq kg^−1^ DW ^137^Cs), the cyanobacterium contained 183 Bq kg^−1^ DW ^134^Cs and 246 Bq kg^−1^ DW ^137^Cs, giving concentration factors of 0.23 for ^134^Cs and 0.26 for ^137^Cs. The concentration factors in the sand were higher than those in the loam soil. The reasons for this difference between soil types should be examined in the future.

The concentration factors for wild *N. commune*, shown in supplementary Table 1, were 0.85–76.01 for ^134^Cs and 0.98–95.89 for ^137^Cs, but were much lower for cultivated *N. commune* (0.23 and 0.62 for ^134^Cs, 0.26 and 0.73 for ^137^Cs). These results suggested that wild *N. commune* directly absorbed radioactively contaminated rainwater. However, *N. commune* absorbed more radioactive cesium from the soil than the seed plants. The ^134^Cs concentration factors were 0.07–0.39 in sunflower and 0.02–0.07 in soybean (5).

Removal of radioactive material from the environment is an urgent problem in affected areas, and soil decontamination using living organisms is being considered. In microbes, cesium-accumulating bacteria have been isolated (15, 16), but collecting these bacteria from polluted soil is difficult. In this study, we demonstrated that the terrestrial cyano-bacterium *N. commune* can absorb high levels of radioactive cesium. Because *N. commune* forms jelly-like clumps, it can be easily collected from the soil surface. Furthermore, its weight decreases by about 90% when *N. commune* is dried. Radiological absorption by *N. commune* may be a viable strategy for decontaminating polluted soil. We will perform decontamination experiments using *N. commune* in the future.

## Supplementary Information



## Figures and Tables

**Table 1:** Radioactivity concentration of ^134^Cs and ^137^Cs in the soil of Iwaki Meisei University, Iwaki City, Fukushima. Soil was collected in April 2012

Depth	^134^Cs (Bq kg^−1^ DW)	^137^Cs (Bq kg^−1^ DW)
0–5 cm	1,630±298^a^	2,530±461
5–10 cm	250±73	424±86
10–15 cm	16±14	31±11
15–20 cm	4±1	5±1
20–25 cm	ND^b^	6±2
25–30 cm	ND	ND

a 3σ counting error.

b not detected.

**Table 2:** Radioactivity concentration of ^134^Cs and ^137^Cs in wild plants and cyanobacterium grown in Iwaki Meisei University, Iwaki City, Fukushima

Species	Collection dates	Shoot		Root	
		^134^Cs (Bq kg^−1^ DW)	^137^Cs (Bq kg^−1^ DW)	^134^Cs (Bq kg^−1^ DW)	^137^Cs (Bq kg^−1^ DW)
**Dicotyledoneae**					
*Artemisia indica var. maximowiczii*	16 April 2012	91±12^a^	184±15	63±9	76±9
*Bidens pilosa var. pilosa*	13 October 2012	55±10	93±11	541±51	1,040±61
*Cerastium glomeratum*	2 May 2012	39±11	59±11	ND^b^	125±28
*Hypochaeris radicata*	2 June 2012	227±16	354±19	192±18	308±21
*Petasites japonicus*	16 April 2012	124±21	174±25	128±12	171±13
*Rumex acetosa*	24 April 2012	93±11	141±13	206±10	314±12
*Solidago canadensis var. scabra*	8 June 2012	54±6	78±7	61±8	100±9
*Veronica persica*	2 May 2012	78±10	136±12	ND	244±64
*Vicia sativa subsp. nigra*	2 May 2012	410±21	557±24	1,120±48	1,520±53
**Monocotyledoneae**					
*Poa annua*	8 May 2012	172±17	219±20	506±39	691±43
*Dactylis glomerata*	15 June 2012	29±7	43±7	106±21	182±22
*Imperata cylindrica*	26 June 2012	73±11	129±12	101±17	214±21
**Cyanobacteria**					
*Nostoc commune*	24 April 2012	32,300±101	46,200±125		

a 3σ counting error.

b not detected.

**Table 3:** Radioactivity concentration of ^134^Cs and ^137^Cs in *Nostoc commune* grown in polluted soil. *N. commune* was cultivated outdoors for 30 days

Soil type	Soil		*Nostoc commune*		Concentration factor	
	^134^Cs (Bq kg^−1^ DW)	^137^Cs (Bq kg^−1^ DW)	^134^Cs (Bq kg^−1^ DW)	^137^Cs (Bq kg^−1^ DW)	^134^Cs	^137^Cs
Sand	4,980±300^a^	6,070±365	3,100±47	4,420±55	0.62	0.73
Loam	785±48	962±58	183±12	246±14	0.23	0.26

a 3σ counting error.

## References

[1] Chino M, Nakayama H, Nagai H, Terada H, Katata G, Yamazawa H (2011). Preliminary estimation of release amounts of ^131^I and ^137^Cs accidentally discharged from the Fukushima Daiichi Nuclear Power Plant into the atmosphere. J Nucl Sci Technol.

[2] Dushenkov S, Mikheev A, Prokhnevsky A, Ruchko M, Sorochinsky B (1999). Phytoremediation of radiocesium-contaminated soil in the vicinity of Chernobyl, Ukraine. Environ Sci Technol.

[3] Fukuda T, Kino Y, Abe Y (2013). Distribution of artificial radionuclides in abandoned cattle in the evacuation zone of the Fukushima Daiichi Nuclear Power Plant. PLos ONE.

[4] Hiyama A, Nohara C, Kinjo S, Taira W, Gima S, Tanahara A, Otaki JM (2012). The biological impacts of the Fukushima nuclear accident on the pale grass blue butterfly. Sci Rep.

[5] Massas I, Skarlou V, Haidouti C (2002). Plant uptake of ^134^Cs in relation to soil properties and time. J Environ Radioact.

[6] Ministry of Agriculture, Forestry and Fisheries of Japan (2011). Removal of radioactive material from farmland soil.

[7] Oshita S, Kawagoe Y, Yasunaga E, Takata D, Nakanishi TM, Tanoi K, Makino Y, Sasaki H (2011). Radioactivity measurement of soil and vegetables contaminated from low level radioactive fall out arised from Fukushima Daiichi Nuclear accident: A study on Institute for Sustainable Agro-Ecosystem Services, Graduate School of Agricultural and Life Sciences, The University of Tokyo. Radioisotopes.

[8] Potts M (1999). Mechanisms of desiccation tolerance in cyanobacteria. Eur J Phycol.

[9] Sakamoto F, Ohnuki T, Kozai N, Igarashi S, Yamasaki S, Yoshida Z, Tanaka S (2012). Local area distribution of fallout radionuclides from Fukushima Daiichi Nuclear Power Plant determined by autoradiography analysis. Trans Atomic Energy Soc Jpn.

[10] Soudek P, Valenová Š, Vavŕíková Z, Vaněk T (2006). ^137^Cs and ^90^Sr uptake by sunflower cultivated under hydroponic conditions. J Environ Radioact.

[11] Staunton S, Dumat C, Zsolnay A (2002). Possible role of organic matter in radiocaesium adsorption in soils. J Environ Radioact.

[12] Takeyasu M, Nakano M, Fujita H, Nakada A, Watanabe H, Sumiya S, Furuta S (2012). Results of environmental radiation monitoring at the Nuclear Fuel Cycle Engineering Laboratories, JAEA, following the Fukushima Daiichi Nuclear Power Plant accident. J Nucl Sci Technol.

[13] Tanoi K, Hashimoto K, Sakurai K, Nihei N, Ono Y, Nakanishi TM (2011). An Imaging of radioactivity and determination of Cs-134 and Cs-137 in wheat tissue grown in Fukushima. Radioisotopes.

[14] Tokyo Electric Power Company (2012). Estimation of the amount of radioactive material release to the atmosphere in the Fukushima Daiichi Nuclear Power Plant accident.

[15] Tomioka N, Uchiyama H, Yagi O (1992). Isolation and characterization of cesium-accumulating bacteria. Appl Environ Microbiol.

[16] Tomioka N, Uchiyama H, Yagi O (1994). Cesium accumulation and growth characteristics of *Rhodococcus erythropolis* CS98 and *Rhodococcus* sp. Strain CS402. Appl Environ Microbiol.

