# Supplementary material for: Accumulation of Radioactive Cesium Released from Fukushima Daiichi Nuclear Power Plant in Terrestrial Cyanobacteria Nostoc commune
Source: Microbes Environ. 2013 Nov 19;28(4):466–9. doi: 10.1264/jsme2.ME13035 (PMC4070698; doi:10.1264/jsme2.ME13035)
Supplement: Supplementary file 1 [file 28_466_s1.pdf]

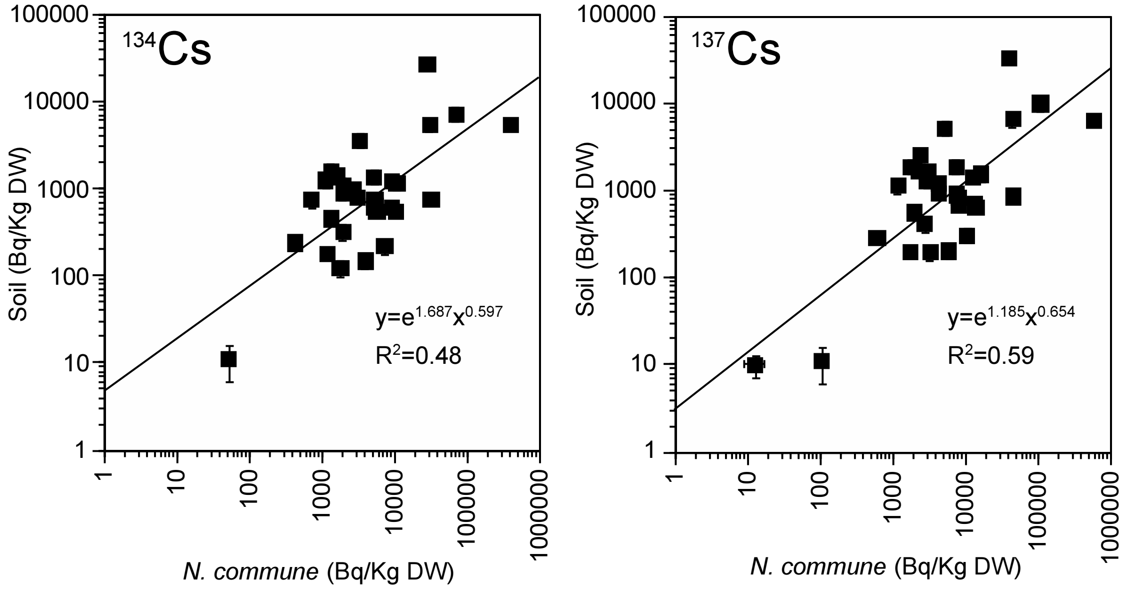

Supplementary Fig. 1.　Correlation of radioactive cesium between *Nostoc commune* and soil.　Bar indicates ± 3σ counting error.

Supplementary Table 1. Radioactivity concentration of ^{134}Cs and ^{137}Cs in *Nostoc commune* and soil. We gathered the soil within 5 cm depth for the measurement.

Collection sites	Collection dates	*Nostoc commune*		Soil	
		^{134}Cs (Bq/kg DW)	^{137}Cs (Bq/kg DW)	^{134}Cs (Bq/kg DW)	^{137}Cs (Bq/kg DW)
Daisen City, Akita	22 August 2012	52 ± 8*	108 ± 9	11 ± 5	11 ± 5
Watari Town, Miyagi (1)	24 August 2011	5750 ± 60	8060 ± 70	543 ± 33	664 ± 41
Watari Town, Miyagi (2)	19 August 2012	725 ± 37	1180 ± 46	738 ± 136	1130 ± 207
Iwaki City, Fukushima (1)	24 August 2011	9380 ± 70	13000 ± 81	1190 ± 72	1410 ± 85
Iwaki City, Fukushima (2)	25 August 2011	10400 ± 111	14000 ± 127	1190 ± 72	1410 ± 85
Iwaki City, Fukushima (3)	25 February 2012	4020 ± 84	5950 ± 100	147 ± 29	202 ± 39
Iwaki City, Fukushima (4)	13 May 2012	5440 ± 51	8020 ± 62	618 ± 114	844 ± 154
Iwaki City, Fukushima (5)	13 May 2012	1970 ± 33	2770 ± 39	319 ± 62	409 ± 78
Iwaki City, Fukushima (6)	31 May 2012	10900 ± 59	16600 ± 74	1140 ± 207	1530 ± 278
Iwaki City, Fukushima (7)	28 August 2012	1980 ± 44	3120 ± 53	1080 ± 195	1620 ± 294
Iwaki City, Fukushima (8)	28 August 2012	1140 ± 33	1810 ± 40	1240 ± 224	1840 ± 333
Iwaki City, Fukushima (9)	28 August 2012	1970 ± 41	3010 ± 50	877 ± 160	1300 ± 236
Iwaki City, Fukushima (10)	24 October 2012	1350 ± 25	2440 ± 34	1590 ± 289	2500 ± 453
Tamura City, Fukushima	4 December 2011	31300 ± 206	45100 ± 243	5300 ± 959	6540 ± 1180
Miharu Town, Fukushima	31 July 2012	3370 ± 60	5300 ± 74	3540 ± 633	5150 ± 931
Koriyama City, Fukushima (1)	19 November 2011	28300 ± 256	39800 ± 297	26500 ± 1590	33200 ± 2000
Koriyama City, Fukushima (2)	30 April 2012	7440 ± 73	10600 ± 87	216 ± 41	303 ± 57
Koriyama City, Fukushima (3)	2 June 2012	71600 ± 452	110000 ± 542	7080 ± 1280	9910 ± 1790
Nihonmatu City, Fukushima	13 August 2011	415000 ± 397	607000 ± 489	5460 ± 329	6330 ± 381
Fukushima City, Fukushima	6 May 2012	5310 ± 122	7580 ± 141	1350 ± 246	1870 ± 339
Date City, Fukushima	6 September 2012	1830 ± 163	3280 ± 192	121 ± 24	194 ± 37
Koga City, Ibaraki	11 September 2011	1210 ± 24	1750 ± 29	178 ± 11	198 ± 13
Yuki City, Ibaraki	6 October 2011	3200 ± 44	4370 ± 51	778 ± 48	911 ± 56
Yachiyo Town, Ibaraki	19 September 2011	1370 ± 32	2000 ± 38	449 ± 28	556 ± 34
Chikusei City, Ibaraki	2 November 2011	1660 ± 27	2370 ± 33	1380 ± 83	1690 ± 102
Oyama City, Tochigi	16 October 2011	2800 ± 38	4220 ± 47	979 ± 59	1210 ± 73
Abiko City, Chiba (1)	29 October 2011	5380 ± 56	7590 ± 66	748 ± 46	900 ± 55
Abiko City, Chiba (2)	29 October 2011	9410 ± 115	13500 ± 134	601 ± 37	719 ± 44
Hino City, Tokyo	15 April 2012	422 ± 22	616 ± 26	238 ± 15	286 ± 18

Kyoto City, Kyoto	3 May 2013	18 ± 4	13 ± 4	ND**	10 ± 3
Osaka City, Osaka	10 September 2011	ND	ND	ND	ND
Mine City, Yamaguchi	15 September 2012	ND	ND	ND	ND
Shimonoseki City, Yamaguchi	14 September 2012	ND	ND	ND	ND
Kitakyushu City, Fukuoka	14 September 2012	ND	ND	ND	ND

*3σ counting error **not detected.
